# Monitoring Protein Denaturation of Egg White Using Passive Microwave Radiometry (MWR)

**DOI:** 10.3390/diagnostics12061498

**Published:** 2022-06-19

**Authors:** Igor Goryanin, Lev Ovchinnikov, Sergey Vesnin, Yuri Ivanov

**Affiliations:** 1 Okinawa Institute of Science and Technology, Onna-son, Okinawa 904-049, Japan; 2 School of Informatics, University of Edinburgh, Edinburgh EH8 9YL, UK; 3 Institute Experimental and Theoretical Biophysics, 142290 Pushchino, Russia; 4 Medical Microwave Radiometry (MMWR) LTD, Edinburgh EH10 5LZ, UK; ksigne@yandex.ru (L.O.); vesnin47@gmail.com (S.V.); 5 Institute of Biomedical Chemistry, 10, Pogodinskaya st., 119121 Moscow, Russia; yurii.ivanov.nata@gmail.com

**Keywords:** microwave radiometry, passive microwave emission, protein denaturation, enzyme assays, drug discovery, brain trauma

## Abstract

Passive microwave radiometry (MWR) is a measurement technique based on the detection of passive radiation in the microwave spectrum of different objects. When in equilibrium, this radiation is known to be proportional to the thermodynamic temperature of an emitting body. We hypothesize that living systems feature other mechanisms of emission that are based on protein unfolding and water rotational transitions. To understand the nature of these emissions, microwave radiometry was used in several in vitro experiments. In our study, we performed pilot measurements of microwave emissions from egg whites during denaturation induced by ethanol. Egg whites comprise 10% proteins, such as albumins, mucoproteins, and globulins. We observed a novel phenomenon: microwave emissions changed without a corresponding change in the water’s thermodynamic temperature. We also found striking differences between microwave emissions and thermodynamic temperature kinetics. Therefore, we hypothesize that these two processes are unrelated, contrary to what was thought before. It is known that some pathologies such as stroke or brain trauma feature increased microwave emissions. We hypothesize that this phenomenon originates from protein denaturation and is not related to the thermodynamic temperature. As such, our findings could explain the reason for the increase in microwave emissions after trauma and post mortem for the first time. These findings could be used for the development of novel diagnostics methods. The MWR method is inexpensive and does not require fluorescent or radioactive labels. It can be used in different areas of basic and applied pharmaceutical research, including in kinetics studies in biomedicine.

## 1. Introduction

Microwave radiometry is currently used in medical studies to measure natural microwave radiation from human tissues [1,2,3]. Commercially available microwave radiometers have been applied in a variety of clinical applications, such as in breast cancer [4], cerebrovascular diseases [5,6], and carotid artery pathology [7], among others.

Human tissues, proteins, and enzymatic solutions emit electromagnetic radiation across a wide frequency range, including the microwave range. This intrinsic electromagnetic radiation can be adsorbed by another object, such as a probe antenna. If there is no reflection between the emitting object and the antenna, i.e., the probe is matched, and there is no additional loss between those bodies, then the power of the electric signal at the probe output will be proportional to the temperature of the emitting object (Equation (1))

(1) $$P=kT_{br}\Delta f$$

where *P*—electric noise power at the output of probe; *T_br_*—brightness temperature; *k*—Boltzmann constant; ∆*f*—receiver bandwidth.

**Figure 1:**
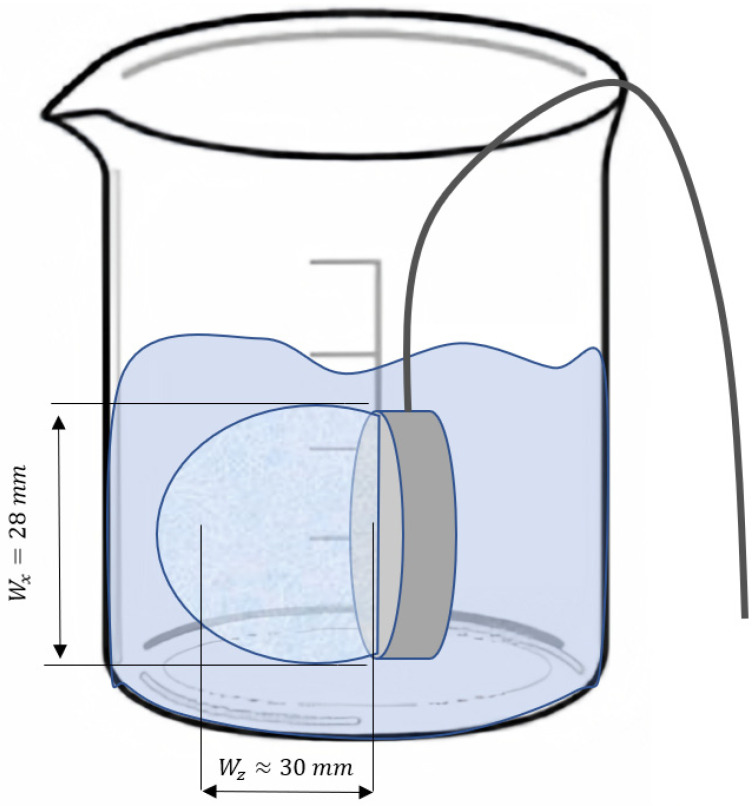
The experimental setup and volume of measurement. A probe is immersed into a liquid. The averaging volume is 28 mm in width (denoted $W_{x}$) and approximately 30 mm in depth (denoted $W_{z}$). Microwave emissions from the liquid are measured.

In thermodynamic equilibrium, the brightness temperature (*T_br_*) is the thermodynamic temperature of a matched black body that produces the same power of radiation that a measured body does. *T_br_* is known to be dependent on probe characteristics and tissue dielectric properties. In principle, the brightness temperature is roughly the average temperature in volume in front of the probe (Figure 1). Volume averaging can be calculated by modeling the electromagnetic interaction between the measured media and the antenna. In passive microwave radiometry, the shape of a measurement volume is generally a hemisphere, and the dimensions can be significantly different. The z-axes (orthogonal to the antenna’s surface) are mostly dependent on the dielectric properties and frequency of the measured media, while the x-axes and y-axes are mostly dependent on the antenna’s dimensions. More details are provided in specialized publications [8].

It is possible to noninvasively obtain information about the averaged thermodynamic temperature by measuring the power of the natural radiation. The area in which a probe receives electromagnetic radiation depends on the wavelength of the received signal. At a wavelength of 30 cm, the depth of measurement is 4–7 cm, depending on the moisture content of the tissues. At a wavelength of 10 cm, the depth of measurement is 2–5 cm^3^. At an ambient temperature of 36 °C and a bandwidth of 0.8 GHz, the power of the received signals is about 3 × 10^−12^ W. This value is commensurate with the level of intrinsic noise of the receiving device and requires special methods to receive and process microwave signals.

Since the last century, thermal imagers or infrared thermographs have been used in medicine [9]. These devices measure temperature based on the radiation of tissues in the infrared (IR) wavelength range. An important difference is that with MWR, it is possible to average temperatures up to 70 mm, allowing the detection of deeper processes, while IR sensors can only measure the temperature of a surface.

Previous experiments featured passive-mode measurements of the MWR of cytochrome P450CYP102A1(BM3) solution during hydroxylation reactions [10]. During another experiment, MWR measurements of a peroxidase reaction in solution, with and without excitation of the solution, were performed [11]. Recently [12], MWR was applied to measure the kinetic rate of bovine serum albumin (BSA) denaturation. 

Human serum albumin is the most abundant protein in blood plasma and is very well-studied. HSA is involved in important biological processes such as osmotic blood pressure, transport, and the metabolism of small molecules and drugs. Additionally, HSA is used as a biomarker for diagnostics and for the treatment of many diseases, such as hypoalbuminemia [13]. HSA consists of 585 amino acids and has a complex globular structure. It is known that during the heating or exposure to certain chemical agents, HSA can unfold and lose its globular structure, i.e., be denaturated. It is then accompanied by significant changes in physical properties.

The kinetics of albumin denaturation have been studied using a variety of methods, including calorimetric studies, absorption spectroscopy, CD spectroscopy, and fluorescence spectroscopy using thermal exposure as well as chemical exposure [14,15,16]. It has been found that temperature exposure causes both denaturation and protein aggregation. The effects of alcohols have been studied [17] by resonance light scattering (RLS), fluorescence spectroscopy, ultraviolet spectrophotometry (UV), circular dichroism (CD), and transmission electron microscopy (TEM). It was found that an ethanol concentration of more than 30% changes the conformation of the protein, making it more hydrophobic, i.e., causing its denaturation. 

Traditional biochemical methods are based on electrochemistry, spectroscopy, or calorimetry; while widely used, they have some disadvantages. Electrochemical methods are characterized by the insufficient stability of the results associated with the state of the electrodes. Circular dichroism requires expensive spectral equipment. Fluorescent methods (which often use fluorescence of aromatic protein groups or labels) can provide additional information about the details of the denaturation process; however, in this case, more expensive fluorimetry is required. Fluorometric systems are usually quite expensive due to the use of both an optical radiation excitation scheme and a real-time recording circuit in the same device. The traditional calorimetry process is a time-consuming technique. Since almost all biological processes are accompanied by thermal processes, it is difficult to relate them to a specific process or phenomenon. 

**Figure 2:**
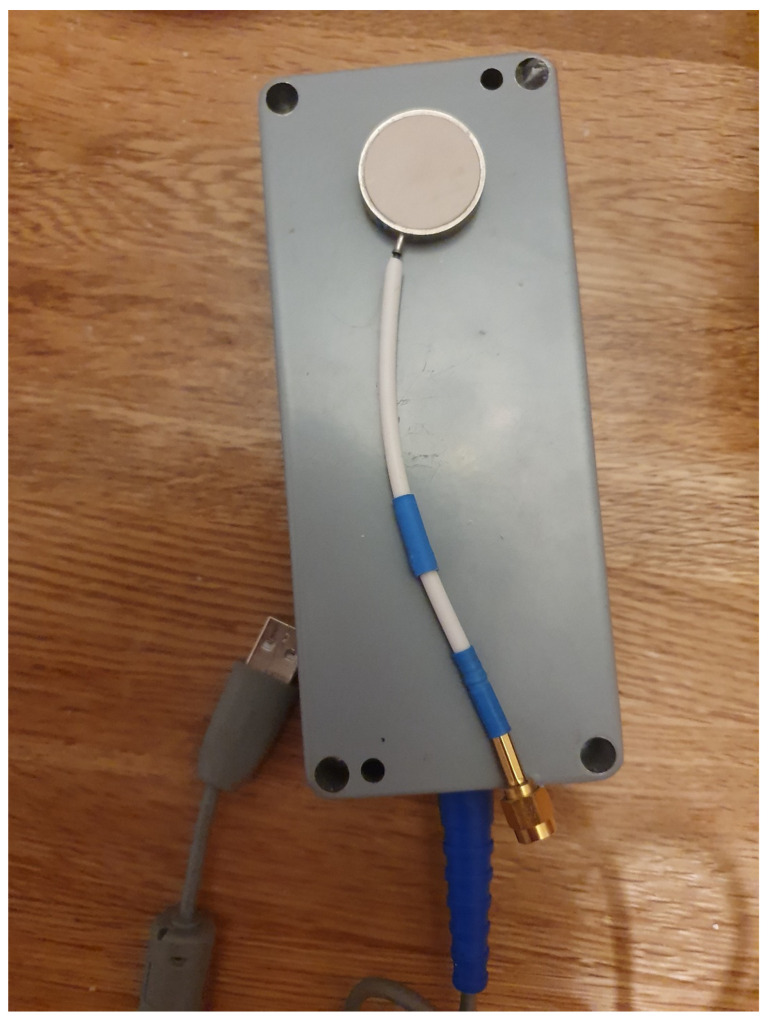
RTMM device with USB connector.

The presented experiment is a continuation of our albumin denaturation experiment [12] and is easy to reproduce. We use an RTMM radiometer (Medical Microwave Radiometry LTD, UK, www.mmwr.co.uk accessed on 19 May 2022) with a round probe to measure microwave radiation from egg whites during alcohol-induced denaturation (Figure 2). For a more detailed description of the technology, see [8]. 

The RTMM device is a precise null-balancing Dicke radiometer 3.8+/−0.4 GHz with a 0.1 s integration time and a 4 s post-processing averaging time. A special disc antenna of 22 mm in diameter is used; the volume of measurement is usually approximately 28 mm in width and 30 mm in depth. The device was calibrated with a pair of graduated, water-filled thermostats. The device is equipped with the specialized software “Neurotherm”, which is capable of capturing and recording microwave radiation-converted temperatures in °C as a time series, with a resolution of up to 60 points in a second. The RTMM device is connected to a USB port of a PC operating on Windows (version 7.0.0 or higher, Microsoft Inc., Seattle, WA, USA)

A contact infrared thermometer (Temlog20, Elitech Ltd., London, UK) was used to measure thermodynamic temperature Large fresh chicken eggs are required to perform this experiment. 

## 2. Materials and Methods 

**Figure 3:**
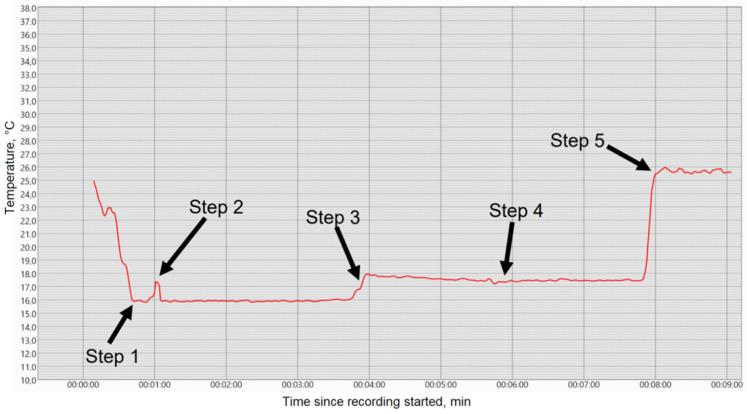
Time dependences were obtained using MWR Dynamics software (MMWR LTD). Step 1. Add egg whites; Step 2. Immerse probe and IR thermometer in the cup; Step 3. Add ethanol 96%; Step 4. Stir; Step 5. Remove probe.

All equipment, water, ethanol, and fresh eggs should be kept at room temperature for at least 6 h so their temperature is uniform and equal to room temperature. The RTMM device should be turned on for at least 20 min before carrying out measurements. The thermometer and the probe should be put inside a cup (volume 70 mL, the height 40 mm). The probe should be put inside the cup with egg white. The probe should be oriented vertically in the cup and positioned at ½ of cup depth. The thermometer should be put inside the cup near the microwave probe but should not touch it. The microwave emission temperature should be monitored using specialized software that can record time series. When there are no more temperature fluctuations <0.3 °C taking place every 2–3 minutes, move to the next step: add 25 mL of 96% ethanol (www.Kelsia.net accessed on 19 May 2022, Aldaia, Spain) to the cup. The sensor and probe are noise protected, but there could be strong external microwave signals (i.e., 5G), which could increase the measurement errors. If there are short peaks, we recommend removing or turning off any mobile phones in the study area. Alternatively, use another location in the room or cover the cup with a metal shield (i.e., aluminum foil). After 5 min, stir the melting mass in the cup with a plastic spoon. Minimize any movement of the probe and continue the recording session for another 5 min. All experiments were performed in triplicate. Using the obtained data, we calculated the slope and determined the kinetics of denaturation, both microwave and thermodynamic, using Equation (1) (Table 1).For control we used 25 mL of tap water instead of ethanol (Figure 3).

## 3. Results

**Figure 4:**
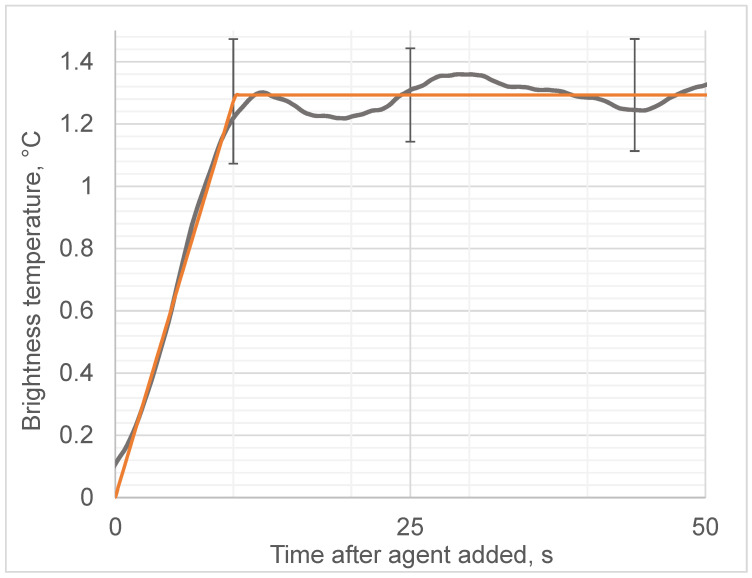
Microwave emissions (brightness) temperature of the egg white during ethanol-induced denaturation. Black line denotes experimentally observed temperature; red line denotes approximation (slope). Different panel figures refer to results from repeated experiments.

**Figure 5:**
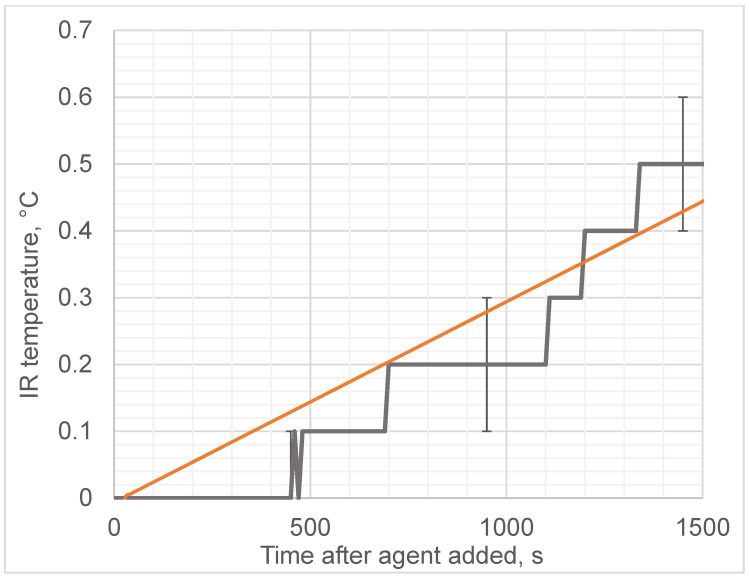
Thermodynamic temperature during alcohol-induced denaturation. Black solid line denotes experimentally observed brightness temperature; orange line denotes approximation (slope).

**Figure 6:**
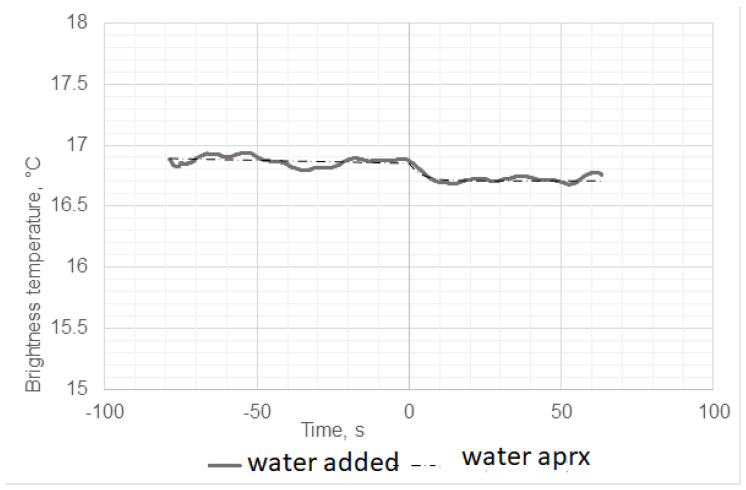
Microwave emissions (brightness temperature) when adding tap water (30 mL) to egg white at Time 0.

**Figure 7:**
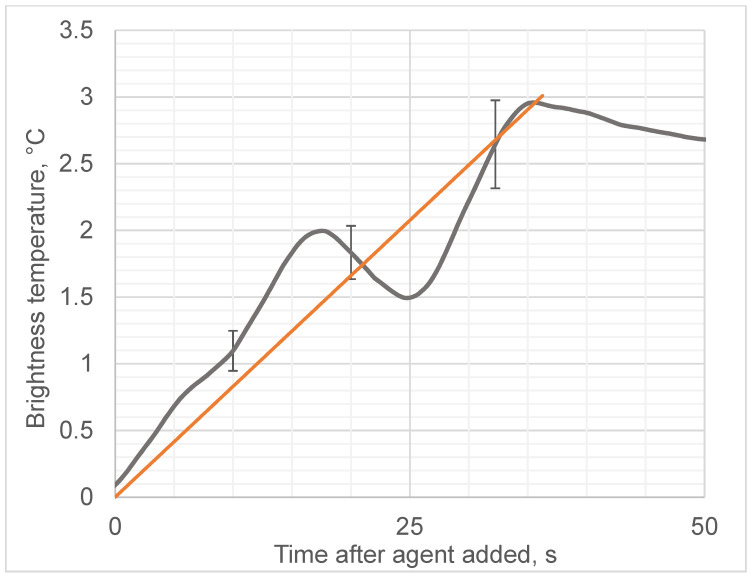
Microwave emissions (brightness temperature) when adding ethanol to tap water during control experiment with no egg white. Black solid line denotes experimental observations. Orange line denotes approximation (slope).

**Figure 8:**
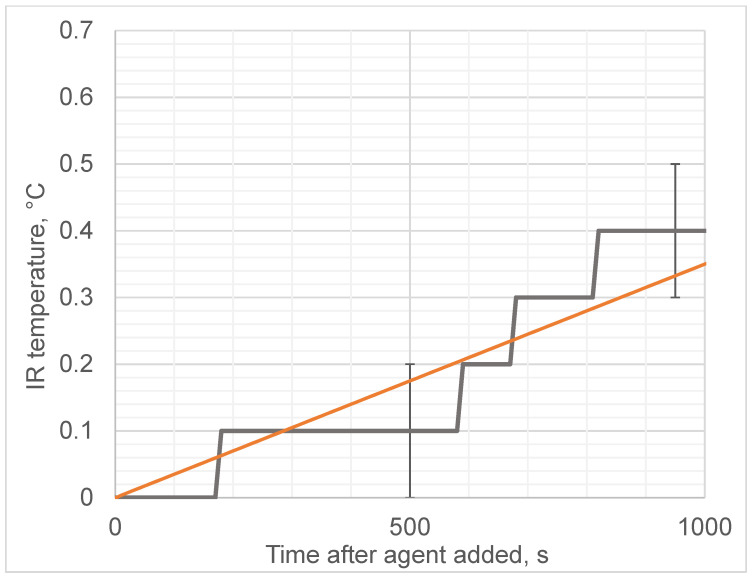
Microwave emissions (brightness temperature) when adding ethanol to tap water during control experiment with no egg white. Black solid line denotes experimental observations. Orange line denotes approximation (slope).

We observed that the addition of ethanol led to a sharp increase in the microwave emissions (brightness temperature) of the solution, as shown in Figure 4, while the thermodynamic temperature increased almost 1000 times more slowly, as shown in Figure 5. In contrast, adding water instead of ethanol to egg whites as a control did not produce any microwave (brightness) or infrared temperature effects (Figure 6). Adding ethanol to water increased MWR and IR temperatures, but at much slower rates in comparison with egg whites (Figure 7 and Figure 8). All of the rate constants are presented in Table 1.

## 4. Discussion

It was shown that microwave emission occurs during egg white temperature denaturation and that the addition of ethanol in this study led to a sharp increase in the microwave radiation of the solution, while the infrared temperature of the solution rises very slowly.

Under the action of a chemical reagent (ethanol), the hydration shell of the protein is destroyed, which leads to the removal of steam water molecules rom the protein shell. In this case, (1) water–alcohol structures are formed, and (2) the protein surface increases, which leads to an increase in the exposure of the hydrophobic groups to the solution. This leads to the formation of protein aggregates. Since the system tends to reach equilibrium, processes occur that remove the overpopulation of rotational water levels, including those that occur due to the microwave radiation at ortho–para water transitions. Since this radiation has a quantum mechanical nature, it proceeds quickly (2 °C in ~20 s), and its flow time is determined by Einstein’s constant for spontaneous emission as well as protein denaturation kinetics, which leads to a change in the ratio of ortho and para isomers in water.

The infrared temperature changed rather slowly (0.5 °C for ~5 min) since it was determined by slower solution-heating processes. This temperature is determined by Brownian motion. Note that earlier, it was shown [18,19] that not only chemical but also mechanical excitation can lead to a change in the structure of water and the relationship between ortho and para isomers in water. We have demonstrated that the mechanical excitation of a fluid near singular points can lead to microwave radiation from water [20]. 

During denaturation processes, complex permittivity can be changed by adding ethanol or other substances. However, since the antenna was matched with the water under investigation, in our opinion, a mismatch in the system caused by the addition of alcohol should lead to an increase in higher frequencies (>10 GHz) and, accordingly, to a decrease in the measured brightness temperature in the microwave range due to a change in the complex permittivity. However, we also observed its increase, which indicates a significant role of the quantum-mechanical processes discussed above.

The effect of microwave emissions could be explained using the concept of molecule rotation in aqueous solutions and the quantum differences between the ortho/para isomers in H_2_O [18]. In the aqueous solutions of proteins, the selective interaction of the para isomers in water with biomolecules leads to an increase in the concentration of ortho isomers. During the conformation of the protein globule, the contact area of this globule with the molecules of the surrounding aqueous medium changes, which, in turn, leads to a shift in the ortho/para–H_2_O ratio. During denaturation, the equilibrium of the ortho/para–H_2_O ratio shifts, and energy is released. This is accompanied by a sharp increase in microwave radiation.

This work demonstrates that MWR allows for the direct monitoring of alcohol-induced protein denaturation without the use of any labels. The process of microwave emission during denaturation is different from emission in the infrared spectrum measured by a thermometer. 

Analysis of the functional status of cells is usually determined using traditional molecular biology methods, such as histological methods, the fluorescent method for determining damage to the plasma membrane of a cell, and electrophysiological methods. These methods are not very convenient and require either various dyes or electrical equipment. The resulting readings are usually not stable due to the influence of buffer salts. To study biological processes, one can distinguish methods based on Raman spectroscopy [21,22]. 

Recently, coherent four-photon scattering (FPS) spectroscopy methods have emerged. These methods allow the investigation of the properties of biological objects based on the registration of optical transitions in the range of ώ1–ώ2~0.1–100 cm^−1^, which makes it possible to record the low-frequency region of the spectrum due to phasing [23]. Phasing is realized in the macroscopic volume of molecular motions using two laser waves with the frequencies ώ1 and ώ2, the difference between which (ώ1–ώ2) is scanned in a wide spectrum in the near-infrared to microwave range. The frequency range from 0.1 to 1 cm^−1^ (corresponding to rotational transitions of water and being in the microwave range) was investigated to study the microenvironment of the proteins and DNA in aqueous solutions [23,24]. 

Other biological objects, including alive cells, can also be studied using MWR, which is simpler, less expensive, and convenient in analytical biochemistry. 

MWR methods provide additional information on biochemical processes, including the denaturation process, by radiation in the radio range, which may be associated with quantum-mechanical transitions in the biological system.

The MWR method is low cost, easy to use, and can produce measurements without using optical and radioactive labels. It does not need to immobilize molecules on the surface of microchips, and it can be used in real time. The target processes are not only the processes of denaturation, but also the processes associated with radiation during continuous-flow enzymatic reactions [9,10] and cellular processes, including those associated with changes in the status of cancer cells [25].

As previously mentioned, the measurements should take place in a location where there is no high microwave noise. The probe should be completely immersed in the egg white. The experiment was performed at room temperature, but the device can work at a wider range of temperatures (5–60 °C). Smaller probes can be used, but as the device measures the volume emissions, the power of the signal should be higher. Therefore, a low-noise microwave room or good microwave noise insulation is required to perform these experiments. The second limitation of the method could be the time interval of the kinetic parameters of denaturation.

Unfortunately, in the current stage of development, MWR cannot separate different processes or indicate the specific mode of a process. 

So far, nobody has been able to explain the effect of increased post mortem microwave emission in the liver [26].

The microwave emission mechanism increases after traumatic brain injuries [27] as well as ischemic [28] and hemorrhagic strokes [29]. Neurogenic fever often develops, significantly worsening the prognosis and outcomes of the disease. We hypothesize that this phenomenon is caused by protein denaturation and protein deficiency in the blood. For example, as an emergency therapy, albumin has been used in patients with cirrhosis and ascites [30] and in patients with acute ischemic stroke [31] for many years. These results could be used for the further development of MWR-based diagnostics systems.

## Figures and Tables

**Table 1:** Rate constants.

Experiment	Linear Slope (MWR), K/s	Linear Slope (IR), K/s
Egg and alcohol	0.127 ± 0.007	3.5 × 10^−4^ ± 0.7 × 10^−4^
Egg and water	−7.5 × 10^−3^ ± 1.5 × 10^−3^	2.5 × 10^−3^ ± 0.2 × 10^−3^
Water and alcohol	0.073 ± 0.01	0.015 ± 0.005
